# Estimating cost-effectiveness in public health: a summary of modelling and valuation methods

**DOI:** 10.1186/2191-1991-2-17

**Published:** 2012-09-03

**Authors:** Kevin Marsh, Ceri J Phillips, Richard Fordham, Evelina Bertranou, Janine Hale

**Affiliations:** 1 United Biosource Corporation, London, W6 7HA, UK; 2 Swansea Centre for Health Economics, Swansea University, Swansea SA2 8PP, UK; 3 Faculty of Health, University of East Anglia, Norwich, NR4 7TJ, UK; 4 TMKG, London, EC1Y 1AA, UK; 5 Health, Social Services and Children Analytical Team, Welsh Government, Cathays Park, Cardiff, CF10 3NQ, UK

**Keywords:** Economic evaluation, Public health, Modelling, Valuation methods

## Abstract

It is acknowledged that economic evaluation methods as they have been developed for Health Technology Assessment do not capture all the costs and benefits relevant to the assessment of public health interventions. This paper reviews methods that could be employed to measure and value the broader set of benefits generated by public health interventions. It is proposed that two key developments are required if this vision is to be achieved. First, there is a trend to modelling approaches that better capture the effects of public health interventions. This trend needs to continue, and economists need to consider a broader range of modelling techniques than are currently employed to assess public health interventions. The selection and implementation of alternative modelling techniques should be facilitated by the production of better data on the behavioural outcomes generated by public health interventions. Second, economists are currently exploring a number of valuation paradigms that hold the promise of more appropriate valuation of public health interventions outcomes. These include the capabilities approach and the subjective well-being approach, both of which offer the possibility of broader measures of value than the approaches currently employed by health economists. These developments should not, however, be made by economists alone. These questions, in particular what method should be used to value public health outcomes, require social value judgements that are beyond the capacity of economists. This choice will require consultation with policy makers, and perhaps even the general public. Such collaboration would have the benefit of ensuring that the methods developed are useful for decision makers.

## Background

Healthcare commissioners are faced with the challenge of allocating resources in order to maximise their objectives [1]. To this end they will need to use sound evidence to inform their decisions. Despite the large investments in the production, synthesis and dissemination of evidence on the effectiveness and cost-effectiveness of healthcare interventions by organisations like the National Institute for Health and Clinical Excellence (NICE) and the National Institute for Health Research (NIHR), the use of evidence by healthcare decision makers remains limited [2]. The challenges of evidence-informed decision making are of equal, if not greater importance in the field of public health, for which the evidence base is much less developed when compared with clinical health. A recent review identified about 150 economic evaluations in the field of public health [3], which compares with many thousands of economic evaluation in health more broadly [4]. If public health interventions are to complete for scarce resources, more evidence is required of their costs and benefits.

The Washington Panel in the United States [5] and the National Institute for Health and Clinical Excellence (NICE) in the UK [6] have developed “reference cases” that set out the range of methodologies deemed most appropriate when undertaking an economic evaluation of health interventions. These guidelines, however, were developed in the context of health technology assessment (HTA) and their applicability in developing priorities within public health requires further consideration. For instance, one of the challenges facing the evaluation of public health interventions is that their objectives are not easily captured by the standard criteria employed by health economists, such as the Quality Adjusted Life Year (QALY) gained [7]. Acknowledging these concerns, NICE [8] has supplemented its reference case for HTA by advocating that for public health costs should be measured from a broader public sector perspective, and a cost-consequence analysis (CCA) may be adopted instead of cost-utility analysis (CUA).

How should economists go about capturing this broader perspective required by public health decision makers? Drummond et al. identified the four main challenges to undertaking economic evaluations of public health interventions as [3]: measuring outcomes; identifying inter-sector costs and consequences; valuing effects; and incorporating equity considerations. The remainder of this paper provides an overview of methods for overcoming two of these challenges – measuring and valuing outcomes.

### Measuring public health outcomes

This section considers two key challenges to measuring the impact of public health interventions: attributing short-term impact to the interventions; and the modelling the other impacts of interventions. The effectiveness of many public health or health promotion interventions depends on changes in individual behaviour, thus making outcomes difficult to attribute and generalise [9]. This challenge has been relatively well covered in the existing literature. For instance, Drummond et al. recommend that, where possible, the relative effectiveness of public health interventions should be assessed by randomised controlled trials (RCTs) [3]. If, however, RCTs are absent and cannot be undertaken, they recommend that gaps in the evidence should be filled by evaluations based on natural experiments and non-experimental approaches. Further, techniques that have been developed to analyse non-experimental data should be more extensively used, such as propensity scores, time series analysis of natural experiments and, where appropriate, more sophisticated econometric modelling and structural simulation modelling.

The second challenge, modelling the impacts not captured by effect studies, has not receive as much attention in the public health economics literature. The types of modelling problem faced by the economic evaluation of public health interventions include: estimating how behaviours change over time, such as whether a short-term improvement in diet is likely to be maintained; estimating the long-term health impacts of behaviours, such as the impact of sedentariness on the incidence of heart disease; estimating the impacts of population interactions, such as sexual behaviour; and estimating multiple co-morbidities, such as those associated with alcohol misuse. What techniques should economists use to incorporate these effects into their analyses?

There is little guidance on how to determine the best structure of an economic model [10,11]. There is, however, a growing literature on how the elements of a modelling problem influence the appropriate modelling approach, which provides an indication of how models of public health interventions might be constructed. The literature identifies a number of fundamental features of modelling approaches that can be used to characterise public health modelling problems [12-14]. First, whether the main features of a model change over time (dynamic models) or not (static models). The importance of this distinction for public health modelling can be illustrated using the example of ‘force of infection’. Public health interventions include behavioural interventions and vaccination programmes to combat the spread of infectious diseases. Models of infectious diseases used to estimate the impact of such interventions may adopt a static approach, assuming that the rate at which susceptible individuals become infected is constant. Given that this rate (or force) of infection is a function of the number of interactions, level of infectiousness, and the distribution of the disease in the population, it is often more realistic to adopt a dynamic approach. This enables the rate of infection to change over time, allowing the impact of effects such as herd immunity to be captured by models.

Second, models can be built so that simulations take place at a cohort or aggregate level, or to allow the behaviour of individuals to be tracked separately. Cohort-level models, such as the cohort Markov models often employed by health economists, allocate individuals to compartments, and require that individuals within a compartment, or cohort, are homogenous. Such cohort models are easier and less resource-intensive to construct than individual-level models, but suffer from a number of limitations. First, the homogeneity assumption is not satisfied, for instance, if future model states depends on individuals’ history. For instance, he health impact of an individual quitting smoking will depend on the individual’s historical consumption of tobacco and alcohol. Second, cohort models become very complex if they need to capture multiple co-morbidities, such as those associated with alcohol misuse, which has been linked with multiple health problems.

In response to these challenges with cohort-level models, a number of individual-level modelling techniques have been proposed, including individual level Markov models, Discrete Event Simulation, and agent-based models [15]. Of particular interest to public health economic evaluation is agent-based models, which allows agents to act autonomously with their own behavioural rules. Many public health interventions are designed to influence peoples’ behaviour, such as improving physical activity levels or causing people to quit smoking. Economic evaluation of such interventions will require an understanding of how peoples’ behaviour responds to these interventions, and how these behavioural impacts are maintained, or otherwise, over time. Agent-based models have an important role to play in such assessments.

To date, the modelling approaches adopted to inform the economic evaluation of public health interventions have tended to be relatively simple, perhaps with the exception of some infectious disease modelling [14]. The variety of modelling approaches available can provide a more accurate assessment of the efficiency of public health interventions. The value of more complex modelling approaches has recently been acknowledged by NICE, which has started to request more complex modelling approaches. If these approaches are to be successful, however, it is important that the evidence base available to economists is improved. The conclusion section consider the type of evidence that would support this research agenda.

### Valuing public health outcomes

If economists are to value the broader range of outcomes generated by public health interventions, a new valuation approach will be required. What approach should be adopted? One alternative to capturing multiple, non-health outcomes is to undertake a cost-consequence analysis (CCA). The CCA provides a ‘balance sheet’ of outcomes that policy makers can weigh up against the costs of an intervention. This option is advocated by NICE in its public health methods guidelines [8]. The draw back to a CCA is, however, that it provides no guidance as to how the different outcomes included in the ‘balance sheet’ should be weighed against each other. This is problematic, for instance, when some outcomes show benefits and others show dis-benefits, and it becomes necessary to ask about the relative value of these outcomes.

The remainder of this section describes the following alternatives approaches to valuing the outcomes generated by public health interventions identified in the literature: cost-benefit analysis (CBA) based on preferential valuation techniques; alternative non-preference-based approaches, the capability and subjective well-being (SWB) approaches; and multi-criteria decision analysis (MCDA).

#### Cost benefit analysis

The limitations with the CUA and CCA can be overcome by valuing outcomes monetarily and undertaking a CBA. This is a method commonly employed in environmental economics [16], where a range of approaches have been applied to value policy outcomes, including: travel cost method; hedonic price method; and the contingent valuation (CV) approach [16-18]), with the latter becoming increasingly popular in the economic evaluation of healthcare programmes [19]. The reliability of stated preference approaches have been debated at length in the environmental economics literature. The National Oceanic and Atmospheric Administration (NOAA) convened an eminent panel to assess the CV methodology – a form of stated preference. The panel concluded that this is a valid approach, provided it is done properly [20].

CBA is recommended by the HM Treasury’s Green Book [21] due to its ability to “to take account of the wider social costs and benefits” and provide outcome measures that are directly comparable with intervention costs. CBA is, however, an approach that is relatively rare in the assessment of public health interventions. Drummond et al’s review of economic evaluations of public health identified no CBAs [3]. McDaid and Needle found that only 5% of the public health economic evaluations used CBA [22].

A key challenge to conducting a CBA is generating monetary values of policy outcomes. In the absence of market prices for outcomes, which is often the case when evaluating public policy, the Green Book recommends that either revealed preference or stated preference methods are used to value outcomes. Revealed preference approaches value outcomes using the preference information revealed through existing markets. For example, the value of preventing a fatality (VPF) has been calculated by estimating the compensating differentials for on-the-job risk exposure in labour markets, or the amount that people are willing to pay for products that reduce risks, such as for automobiles and fire alarms [23]. However, the validity of the estimates produced by revealed preference techniques depend on the ability to isolate, for instance, the impact of VPF from the other factors that determine wages and prices. Furthermore, the revealed preference approach is based on the assumption that markets work well. The difficulty in fulfilling these requirements is thought to explain the large variation in estimates produced by revealed preference studies, with, for example, VPF estimates ranging from £0.5 million to £64.0 million [24].

Given the difficulties posed by the revealed preference approach, economists have turned to stated preference approaches to value non-market goods. Stated preference studies construct a hypothetical contingent market where the individual is surveyed to identify their willingness to pay (WTP) for the non-market good. Since the 1990s, the stated preference literature has grown rapidly, especially within environmental economics. Stated preference techniques have been used to identify the relative value of public health outcomes. For instance, a discrete choice experiment (DCE) was undertaken recently to determine the relative values health decision makers in the UK place on the cost-effectiveness, impact on health inequalities, and population reached by public health interventions [25]. The advantage of stated preference approaches is their ability to directly elicit the information that is required. However, stated preference techniques face a number of challenges, with probably the main one being the assumption that individuals have a coherent set of preferences. A number of phenomena have been identified as evidence that such coherent preference may not be observed in practice [24].

#### Alternative notions of well-being

Despite reassurances from organisations like the NOAA, concerns about the challenge facing preference-based approaches described above have caused economists to search for alternative, non-preference-based notions of value. Two alternatives that have recently received most attention are the capability approach and the SWB approach.

Developed by Amartya Sen, the capability approach advocates that programmes are evaluated based on their impact on the extent to which a person is able (has the capability) to function in a particular way [26]. An example of the capability approach being applied in health economics is Birmingham University’s development of a measure of capability wellbeing for adults [27]. This identifies five over-arching attributes of capability wellbeing: stability, attachment, achievement, autonomy and enjoyment. Each attribute is then described using four response categories. In its use of a descriptive system based on dimensions or attributes, the evaluation approach proposed by the capability approach is similar to the QALY approach. The key difference between these evaluation approaches is that the descriptive system employed by the QALY is limited to one dimension – health – and to functioning rather than capability – health status rather than the capability (freedom) to pursue health improvement [26]. Furthermore, the capability approach does advocate a preference-based approach to weight attributes.

The capability approach has been criticised for relying on expert opinion about what contributes to well-being, and an inability to generate a monetary estimate of benefit [28]. Another alternative has been suggested that does not suffer from these limitations – SWB. The SWB approach involves measuring how people’s self-assessment of their well-being varies as they experience outcomes targeted by policy. Well-being assessments are elicited, for instance, in responses to questions such as how satisfied people are with their life overall. Answers are generally recorded on scales ranging from, for instance, 1 for not satisfied at all to 7 for completely satisfied.

The SWB approach to valuing non-market goods is increasingly being employed by economists as an input to public policy making [23]. Furthermore, the possibilities of SWB measures are also being recognised by policy makers themselves. Perhaps most prominent among these initiatives was President Nicolas Sarkozy’s commission into measuring progress, which was chaired by a number of eminent Nobel Prize-winning economists [29], and David Cameron’s request that the Office of National Statistics measure the happiness of population in the UK [30].

There was initial scepticism about whether responses to life satisfaction questions could be sensitive enough to capture the effect of policy outcomes. There is, however, a growing literature on the sensitivity and validity of responses to life satisfaction questions. Responses yield consistent and intuitively appealing associations between well-being and life experiences, such as health and employment [23,31]. Responses have been shown to have a causal relationships with actual behaviour, e.g. suicide, and key physiological variables [28]. Psychological studies showing how those with higher scores are more likely to be rated as happy by friends and less likely to show signs of mental disorder [31].

Thus, the SWB approach is attracting attention as a possible solution to the challenges associated with preference-based measures of value, and could provide a more holistic approach to capturing and valuing the outcomes associated with public health interventions. It is important, however, to note that the approach is still in its infancy and that a number of methodological questions need to be answered before this approach can be used to generate evidence to inform policy making [32].

#### Multi-criteria decision analysis

Multi-criteria decision analysis (MCDA) approaches vary according to the source and nature of information used to inform decision making, but they include four common steps: identifying interventions; identifying evaluation criteria; measuring the interventions against the criteria; and combining the criteria scores using a weighting to produce an overall assessment of each intervention. MCDA approaches have been adopted across a range of policy areas, including by environmental economists [33]. A key feature of MCDA – its ability to capture a range of policy outcomes – is the reason that there has been a recent increase in attention in the approach from amongst health economists interested in capturing a broader value in a more systematic manner [34].

There are already a number of examples of MCDA having been successfully applied to the prioritisation of public health interventions. For instance, the US by the Preventive Services Task Force ranked a list of clinical preventative interventions based on their cost-effectiveness and clinical preventable burden [35,36]. More recently a MCDA approach has been used to prioritise preventative health interventions in England [25,37,38].

MCDA approaches vary according to the source and nature of information used. The range of approaches adopted is referred to as ‘the socio-technical system’, or the balance between decision maker input and researcher measurement. The different components of the MCDA can each be classified as either deliberative or data-driven, depending on the source of data drawn on. Data-driven approaches to weighting/valuing policy outcomes could, for instance, include the preferential and experiential approaches referred to above. Deliberation refers to the process of negotiation between various stakeholders, usually through the use of workshops. There is disagreement as to exactly what balance should be struck between decision maker and researcher input into a MCDA, with the answer to this question being a function of the objective of the MCDA [39]. It is, however, important to acknowledge two key benefits of more deliberative approaches to weighting policy outcomes. First, they are often less time- and resource-intensive. Thus, they can be replicated in different localities, generating locally specific outcome valuations. Second, they engage decision makers in the evaluation process, ensuring their buy-in to the results of the evaluation. This helps address a key challenge of more traditional HTA – the utilisation of economic evaluation evidence by decision-makers [2].

## Conclusion

The evaluation of the efficiency of public health interventions, both relative to each other and to other health and social care interventions, is necessary if the maximum benefits are to be derived from the use of public resources. This paper considers the methods required to better measure and value the outcomes generated by public health interventions. We propose two areas of method development as being priority.

First, more work is required to determine the appropriate methods for, and to provide data to support, the modelling of behavioural outcomes and the link between these outcomes and health and other outcomes. We welcome the move to more complex modelling approaches of behaviour in the economic analysis of public health interventions. These approaches offer the potential to provide a more accurate assessment of the efficiency of public health interventions. Two research initiatives would facilitate the development of these models. Further work is required to determine the most appropriate types of model for different behavioural outcomes. Health economists often adopt Markov models to simulate intervention effects. It is, however, recognised within HTA that Markov models are not always the most appropriate way to model health outcomes. Alternatives, such as Discrete Event Simulation, are proposed as more appropriate in certain circumstances. It is important that economists working in public health consider the relative merits of these alternative modelling approaches, rather than simply adopting the conventions that have, at least so far, served HTA well.

The selection and implementation of appropriate modelling approaches will be supported by the development of a better empirical understanding of behavioural dynamics and the relationship of behaviour and health. For instance, existing panel survey data should be analysed to determine the dynamics of relevant public health behaviours, such as physical activity, diet, smoking, alcohol consumption, and sexual behaviour. This evidence should also be analysed to determine the impact of these behaviours on health outcomes, by age and accumulated behaviour.

Second, further research should be undertaken into how to value public health outcomes. The wide range of outcomes generated by public health interventions cannot be captured by the valuation techniques conventionally used in HTA. One response to this challenge would be to adopt the approaches of economist working in other policy areas, such as the environment, and use revealed and stated preference approaches to value outcomes monetarily and undertake a CBA. These approaches, however, face a number of methodological challenges. Beyond these methods considerations, it is also important to ask whether the nature of the valuations implicit in these approaches is desirable. First, both stated and revealed preference approaches base valuation on population preferences. Economists have been exploring the possibility of alternative approaches based on population experiences or capabilities. Second, valuation approaches need to be transparent and acceptable to the decision makers using the outcome of economic evaluations. Given these alternatives, we agree with Drummond et al. [3] that there should be more debate about the value propositions underlying the various forms of economic evaluation, and their appropriateness for assessing public health interventions. Furthermore, this debate needs to involve the decision makers using the outputs from economic research.

## Competing interests

The authors have no conflicts of interest.

## Authors’ contribution

K Marsh led the review and the writing of the paper. E Bertranou conducted searches to support the review, and summarised contents of key articles. C Phillips and R Fordham contributed to drafting sections of the paper, and provided extensive comments on the paper. J Hale provided recommendations for papers to include in the review, and extensive comments on the paper. All authors read and approved the final manuscript.
